# Parental Broflanilide Exposure Impairs Offspring Fitness and Alters Detoxification Enzymes and Gut Microbiota in *Helicoverpa armigera*

**DOI:** 10.3390/insects17080878

**Published:** 2026-08-21

**Authors:** Xue Gao, Qihui Li, Fangfang Yan, Chenyu Su, Xiufang Wang, Pengjun Xu, Guangwei Ren, Min Mao

**Affiliations:** 1 Key Laboratory of Tobacco Pest Monitoring Controlling & Integrated Management, Tobacco Research Institute, Chinese Academy of Agricultural Sciences, Qingdao 266101, China; 2 Panzhihua Branch of Sichuan Tobacco Company, Panzhihua 617026, China

**Keywords:** sublethal effect, life table, detoxification metabolism, bacterial community, intergenerational effect

## Abstract

The cotton bollworm *Helicoverpa armigera* (Hübner, 1808) (Lepidoptera: Noctuidae) is a globally distributed polyphagous pest that damages various crops. Pesticides remain a primary and effective strategy for controlling cotton bollworm populations. This study evaluated the effects of broflanilide on *H. armigera* by integrating life-table analysis, detoxification enzyme activity assays, and gut microbiome characterization. Broflanilide showed high toxicity against third-instar larvae. Parental LC_50_ exposure prolonged larval development and reduced larval survival in the F_1_ generation. In addition, parental exposure to both LC_30_ and LC_50_ reduced adult emergence, fecundity and major population growth parameters, especially the intrinsic rate of increase (*r*) and net reproductive rate (*R*_0_), demonstrating a significant transgenerational inhibitory effect. Enzyme assays showed that carboxylesterase (CarE) activity was induced after broflanilide exposure, glutathione S-transferase (GST) activity showed a time-dependent response with the strongest induction generally observed under LC_10_ treatment, whereas cytochrome P450 monooxygenase (P450) activity was generally inhibited. Gut microbiota analysis showed that LC_50_ exposure significantly reduced the relative abundance of *Enterococcus*. Functional prediction further indicated enrichment trends in pathways related to xenobiotic degradation and metabolism. Overall, broflanilide not only exhibited strong lethal activity against *H. armigera*, but also produced sustained effects on population fitness, detoxification metabolism and gut microbial ecology. These findings provide a theoretical basis for the rational use of broflanilide and improve our understanding of its sublethal physiological and microbial effects on *H. armigera*.

## Simple Summary

The cotton bollworm is an important agricultural pest whose larvae feed on more than 200 crop species, including cotton, maize, wheat and tobacco, causing substantial economic losses worldwide. Its management currently relies predominantly on chemical insecticides; however, this pest has developed resistance to numerous conventional insecticides. Broflanilide is a novel insecticide with a distinct mode of action. This study investigated the effects of broflanilide exposure on the growth, development, reproduction, detoxification enzymes and gut microbiota of the cotton bollworm. The results showed that parental exposure to broflanilide delayed offspring development and reduced adult fecundity, leading to a marked decline in population growth potential. Exposure also altered detoxification enzyme activities and affected the relative abundance of specific gut bacterial taxa. However, the direct roles of these enzymes and bacterial taxa in broflanilide metabolism require further functional validation. These findings indicate that broflanilide exerts both direct and intergenerational suppression of this pest. They provide a scientific basis for the rational application of this insecticide and contribute to safeguarding crop yields and food security.

## 1. Introduction

The cotton bollworm, *Helicoverpa armigera* (Hübner, 1808) (Lepidoptera: Noctuidae), is a globally distributed polyphagous lepidopteran pest that feeds on more than 200 crop species, including cotton, maize, wheat and tobacco. It occurs widely in agricultural systems in China and causes substantial economic losses [1,2]. Chemical control remains a major strategy for managing *H. armigera*. However, long-term and frequent insecticide use has led to varying levels of resistance to multiple classes of insecticides [3,4,5]. For example, resistance ratios to pyrethroids in some regional populations have exceeded 100-fold, resulting in reduced field efficacy [6,7,8]. Therefore, insecticides with novel modes of action and limited cross-resistance with conventional compounds are important for the sustainable management of *H. armigera* [9].

Broflanilide is a novel meta-diamide insecticide that acts as a negative allosteric modulator of the resistance to dieldrin (RDL) subunit of insect γ-aminobutyric acid (GABA)-gated chloride channels [10]. Because its mode of action differs from those of pyrethroids, neonicotinoids and other diamide insecticides, broflanilide has considerable potential for resistance management. Previous studies have shown that broflanilide has high biological activity against several lepidopteran pests [11,12]. However, existing research has mainly focused on lethal toxicity, whereas the ecotoxicological effects induced by sublethal exposure remain less well understood [13,14]. Under field conditions, pesticide residues, uneven application and pest migration may expose insects to sublethal concentrations over extended periods. Sublethal exposure can affect insect development, survival and reproduction. It may also induce intergenerational effects through maternal resource allocation, endocrine regulation or epigenetic modification, thereby influencing offspring population dynamics [15]. To date, information on the sublethal and intergenerational effects of broflanilide on *H. armigera* remains limited.

The tolerance and metabolism of xenobiotics in insects largely depend on detoxification enzyme systems, including cytochrome P450 monooxygenases (P450s), carboxylesterases (CarEs) and glutathione S-transferases (GSTs). These enzymes play important roles in pesticide metabolism and resistance development [16], and changes in their activities are commonly used to evaluate physiological responses to chemical stress [17]. Therefore, clarifying the effects of broflanilide on the detoxification enzyme system of *H. armigera* may help improve our understanding of its physiological responses to broflanilide exposure.

In recent years, increasing evidence has shown that insect gut microbiota contributes to nutrient metabolism, immune regulation and xenobiotic degradation [18]. Some gut symbionts can directly participate in insecticide degradation or indirectly enhance host tolerance by regulating the expression of detoxification-related genes [19,20]. At the same time, pesticide exposure may disrupt gut microbial homeostasis, alter community structure and further affect host fitness and environmental adaptability [21]. However, the effects of broflanilide on the gut microbial community of *H. armigera* remain largely unexplored.

In this study, we used age-stage, two-sex life-table analysis to evaluate the effects of exposure to the sublethal concentration LC_30_ and the median lethal concentration LC_50_ on development, reproduction and population parameters in the F_0_ and F_1_ generations. In parallel, we measured the dynamic changes in the activities of major detoxification enzymes, including CarE, GST and P450, after exposure to different broflanilide concentrations. We also characterized the gut bacterial community using 16S rRNA high-throughput sequencing. This study aimed to systematically assess the lethal, sublethal and intergenerational ecological effects of broflanilide on *H. armigera* and to explore potential physiological and gut microbial responses, providing a theoretical basis for the rational use of broflanilide.

## 2. Materials and Methods

### 2.1. Insect 

A laboratory colony of *H. armigera* was obtained from the Plant Protection Research Center, Tobacco Research Institute, Chinese Academy of Agricultural Sciences. The colony was originally collected from agricultural fields in Henan Province, China, and had been maintained under laboratory conditions for more than 10 generations without exposure to insecticides. Larvae were reared on an artificial diet [22] at 25 ± 1 °C, 65–75% relative humidity and 14:10 h (L:D) photoperiod. Third-instar larvae from this colony were used for all experiments.

### 2.2. Insecticides

Technical-grade broflanilide (99.51%) was purchased from Bide Pharmatech Co., Ltd. (Shanghai, China). Stock solutions were prepared in acetone and diluted with 0.1% Tween-80 aqueous solution to the required concentrations prior to use. 

### 2.3. Toxicity Bioassay

The toxicity of broflanilide to third-instar *H. armigera* larvae at 72 h was determined using an artificial diet surface-overlay method [12]. The 72-h LC_10_, LC_30_, and LC_50_ values of broflanilide were 0.107, 0.253, and 0.457 mg L^−1^, respectively, and the LC_50_ value of lambda-cyhalothrin was 25.222 mg L^−1^. These values were previously determined by our research team using the same laboratory colony, larval instar, bioassay method, and environmental conditions as in the present study [23]. Based on the reported baseline LC_50_ (0.274 mg L^−1^) of broflanilide to a susceptible colony of *H. armigera* [12], the toxicity ratio of the tested colony was 1.7, indicating that the tested colony remains at a susceptible level.

### 2.4. Life-Table Experiments

Based on the toxicity bioassay, LC_30_ and LC_50_ concentrations of broflanilide were selected for life-table experiments. For each treatment, 120 third-instar larvae were initially exposed to broflanilide. All larvae surviving the 72 h exposure period were included in the subsequent life-table observations. Each surviving larva was individually transferred to 25 mL plastic cups containing fresh artificial diet. Development and survival were recorded daily, and the diet was replaced every 48 h.

Pupae were weighed on the third day after pupation and maintained individually until adult emergence. Newly emerged adults from the same treatment were paired in 500 mL plastic containers and supplied with 10% honey solution. Adult survival and egg production were recorded daily until death. Eggs laid between days 2 and 5 after adult emergence were collected as the F_1_ generation. Newly hatched F_1_ larvae were reared individually under the same conditions, with 120 larvae per treatment.

### 2.5. Detoxification Enzyme Assays

Third-instar larvae were exposed to LC_10_, LC_30_ and LC_50_ concentrations of broflanilide as described above. Control larvae were treated with 0.1% Tween-80 solution. Surviving larvae were collected at 24 and 72 h after exposure. Each biological replicate consisted of 5–8 larvae, and each treatment included three biological replicates.

Enzyme activities were measured using whole-body homogenates of the larvae. Larvae were rinsed with distilled water, blotted dry and weighed. Crude enzyme extracts were prepared according to the instructions of commercial assay kits. Activities of carboxylesterase (CarE), glutathione S-transferase (GST) and cytochrome P450 monooxygenase (P450) were measured using a microplate reader and expressed as units per milligram of protein. The enzyme activity assay kit was provided by Jiangsu Addison Biotechnology Co., Ltd. (Yancheng, China).

### 2.6. Gut Microbiota Analysis

Third-instar larvae exposed to LC_10_ or LC_50_ broflanilide for 72 h were collected for gut microbiota analysis. Larvae treated with 0.1% Tween-80 solution were used as controls. Each treatment included three biological replicates, and each replicate consisted of pooled gut tissues from 10 larvae.

Larvae were anesthetized on ice, surface-sterilized with 75% ethanol and 0.25% sodium hypochlorite, and rinsed three times with sterile distilled water. Gut tissues were dissected under sterile conditions, frozen in liquid nitrogen and stored at −80 °C until DNA extraction.

Total genomic DNA was extracted using the E.Z.N.A.^®^ Soil DNA Kit (Omega Bio-tek, Norcross, GA, USA). DNA quality was checked by 1% agarose gel electrophoresis, and DNA concentration was measured using a QuantiFluor™-ST fluorometer (Promega Corporation, Madison, WI, USA). The V3–V4 region of the bacterial 16S rRNA gene was amplified using primers 338F and 806R. Purified PCR products were sequenced on an Illumina MiSeq PE300 platform (Illumina Inc., San Diego, CA, USA).

Raw reads were quality-filtered using fastp v0.23.4 and merged using FLASH v1.2.11. Amplicon sequence variants (ASVs) were generated using the DADA2 pipeline (2024) and taxonomically assigned against the SILVA database release 138 with a confidence threshold of 0.7 [24].

### 2.7. Statistical Analysis

Developmental duration, pupal weight, pupation rate, adult emergence rate, adult malformation rate, fecundity, oviposition period and detoxification enzyme activities were tested for normality and homogeneity of variance. All datasets met the assumptions of normality and homogeneity of variance and were analyzed using one-way ANOVA followed by Tukey’s honestly significant difference (HSD) test for multiple comparisons. Differences were considered significant at *p* < 0.05.

Age-stage, two-sex life-table parameters were analyzed using TWOSEX-MSChart v2.00.2591 [25]. Standard errors of the intrinsic rate of increase (*r*), finite rate of increase (*λ*), net reproductive rate (*R*_0_) and mean generation time (*T*) were estimated by bootstrap analysis with 100,000 resampling. Differences among treatments were compared using paired bootstrap tests at *p* < 0.05.

For gut microbiota analysis, alpha diversity was evaluated using the Shannon and Simpson indices. Differences among treatments were analyzed by one-way ANOVA followed by Games–Howell post hoc tests, with false discovery rate (FDR) correction for multiple comparisons. Differences were considered significant at FDR-corrected *p* < 0.05. Beta diversity was assessed using weighted UniFrac distances and visualized by principal coordinate analysis (PCoA). Differences in bacterial community structure among treatments were evaluated using ANOSIM with 999 permutations. Differentially abundant bacterial genera between the broflanilide-treated and control groups were identified using ANCOM at the genus level [26]. Functional prediction was performed using PICRUSt2 v2.2.0 based on the KEGG database [27]. Microbial community analyses were conducted in QIIME2 (2024) [28] and R v4.2.0.

## 3. Results

### 3.1. Effects of Sublethal and Median Lethal Broflanilide Exposure on Biological Traits

#### 3.1.1. Biological Traits of the F_0_ and F_1_ Generations

Exposure to broflanilide affected selected biological traits of the F_0_ generation of *H. armigera* (Table 1). Compared with the control, both LC_30_ and LC_50_ exposure significantly prolonged the duration of the fifth and sixth larval instars, from 5.92 d in the control to 7.04 and 7.39 d, respectively (*p* < 0.05). Larval survival was significantly reduced only in the LC_50_ group, decreasing from 90.80% in the control to 39.00%. Neither pupal duration nor pupation rate differed significantly among treatments. Pupal weight differed between the LC_30_ and LC_50_ groups, but neither treatment differed significantly from the control. At the adult stage, LC_50_ exposure significantly reduced the adult emergence rate to 78.52% and increased the adult malformation rate to 15.88%. Reproductive performance was also impaired by LC_50_ exposure, with fecundity decreasing to 1557.50 eggs per female and the oviposition period shortening to 8.06 d.

Although the F_1_ generation was not directly exposed to broflanilide, parental exposure affected several developmental, survival and reproductive traits in the offspring (Table 2). Parental LC_50_ exposure significantly prolonged the duration of the first to fourth larval instars, resulting in a total larval duration of 18.95 d, compared with 14.21 d in the control group (*p* < 0.05). In contrast, the duration of the fifth and sixth instars did not differ significantly among treatments. Larval survival was significantly reduced only in the LC_50_ group, decreasing from 84.00% in the control to 31.19%. Pupal duration, pupal weight and pupation rate were not significantly affected by parental exposure. At the adult stage, emergence rates were significantly reduced in both the LC_30_ and LC_50_ groups, reaching 65.28% and 59.32%, respectively, compared with 97.22% in the control. Adult malformation increased significantly only in the LC_50_ group. Fecundity decreased progressively with parental exposure concentration, from 2117.67 eggs per female in the control to 1546.00 and 1243.63 eggs per female in the LC_30_ and LC_50_ groups, respectively. The oviposition period was significantly shortened in the LC_50_ group.

#### 3.1.2. Population Parameters of the F_1_ Generation

Two-sex life-table analysis revealed significant differences in *r*, *λ*, *R*_0_ and *T* among treatments (Table 3). The intrinsic rate of increase (*r*) was 0.1862 day^−1^ in the control, but decreased to 0.1463 and 0.1261 day^−1^ in the LC_30_ and LC_50_ groups, respectively, representing reductions of 21.4% and 32.3% (*p* < 0.05). The finite rate of increase (*λ*) showed a similar decreasing trend. The net reproductive rate (*R_0_*) decreased to 215.72 and 188.63 offspring per individual in the LC_30_ and LC_50_ groups, respectively, corresponding to reductions of more than 70% relative to the control. The mean generation time (*T*) was significantly prolonged in the LC_50_ group, reaching 41.56 d compared with 35.63 d in the control, whereas no significant difference was observed between the LC_30_ group and the control. Correspondingly, the population doubling time (DT) increased from 3.72 d in the control group to 4.74 d and 5.50 d in the LC_30_ and LC_50_ groups, respectively.

**Figure 1:**
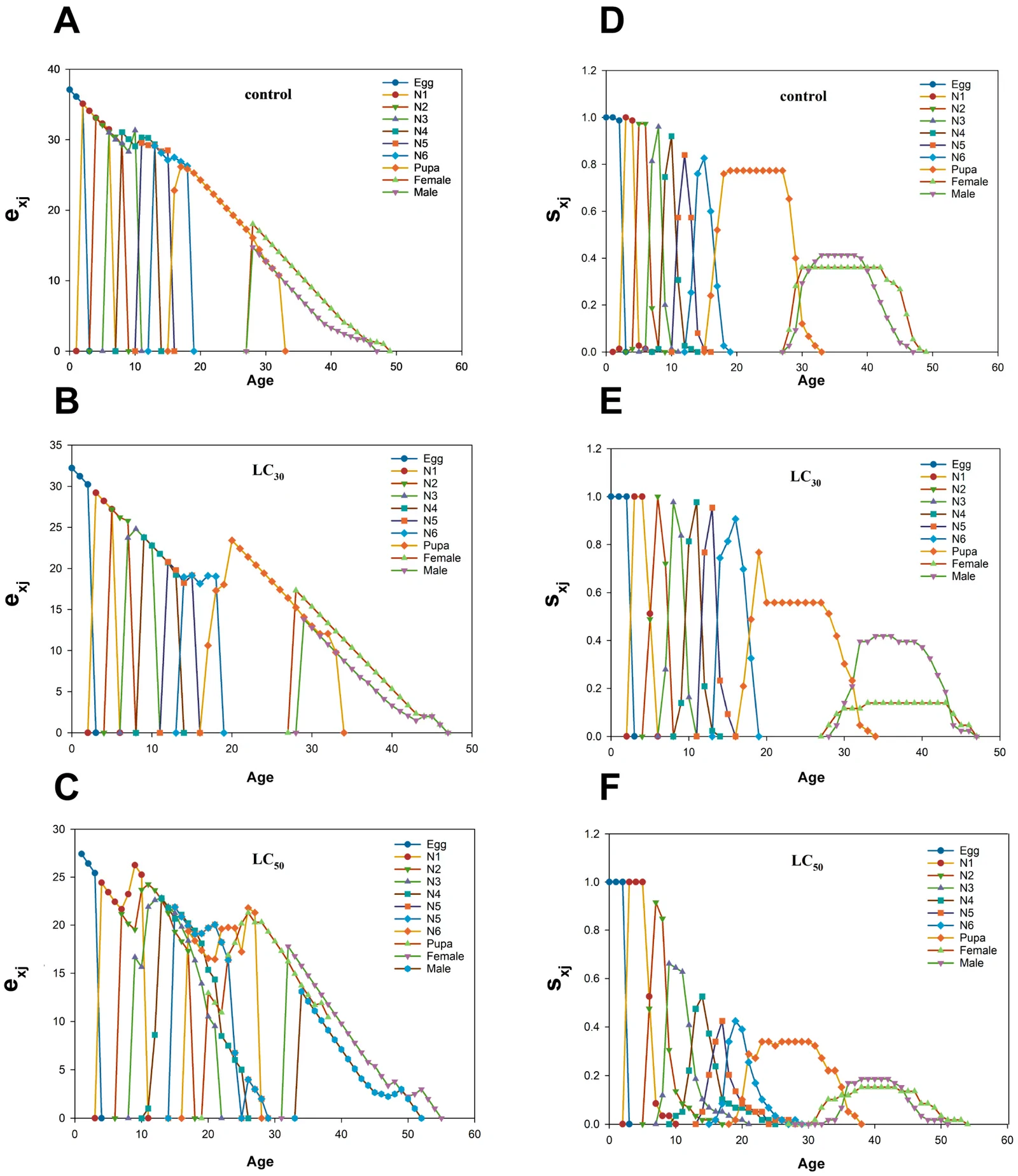
Age-stage specific life expectancy (*e_xj_*) and age-stage survival rate (*s_xj_*) of the F_1_ generation of *Helicoverpa armigera* after parental exposure to broflanilide. (**A**–**C**) Age-stage specific life expectancy (*e_xj_*) curves under control, LC_30_, and LC_50_ treatments, respectively. (**D**–**F**) Age-stage specific survival rate (*s_xj_*) curves under control, LC_30_, and LC_50_ treatments, respectively. N1–N6 represent first- to sixth-instar larvae.

The age-stage-specific life expectancy ($e_{xj}$) at age zero was 37.11, 32.21 and 27.42 d in the control, LC_30_ and LC_50_ groups, respectively (Figure 1A–C). The age-stage-specific survival rate ($s_{xj}$) curves overlapped among developmental stages because of individual variation in developmental rates (Figure 1D–F). The probabilities of surviving from the egg stage to adulthood were 0.773, 0.558 and 0.339 in the control, LC_30_ and LC_50_ groups, respectively. Adults first appeared at age 28 d in both the control and LC_30_ groups and at age 31 d in the LC_50_ group.

### 3.2. Effects of Broflanilide Exposure on CarE, GST and P450 Activities in Helicoverpa armigera

**Figure 2:**
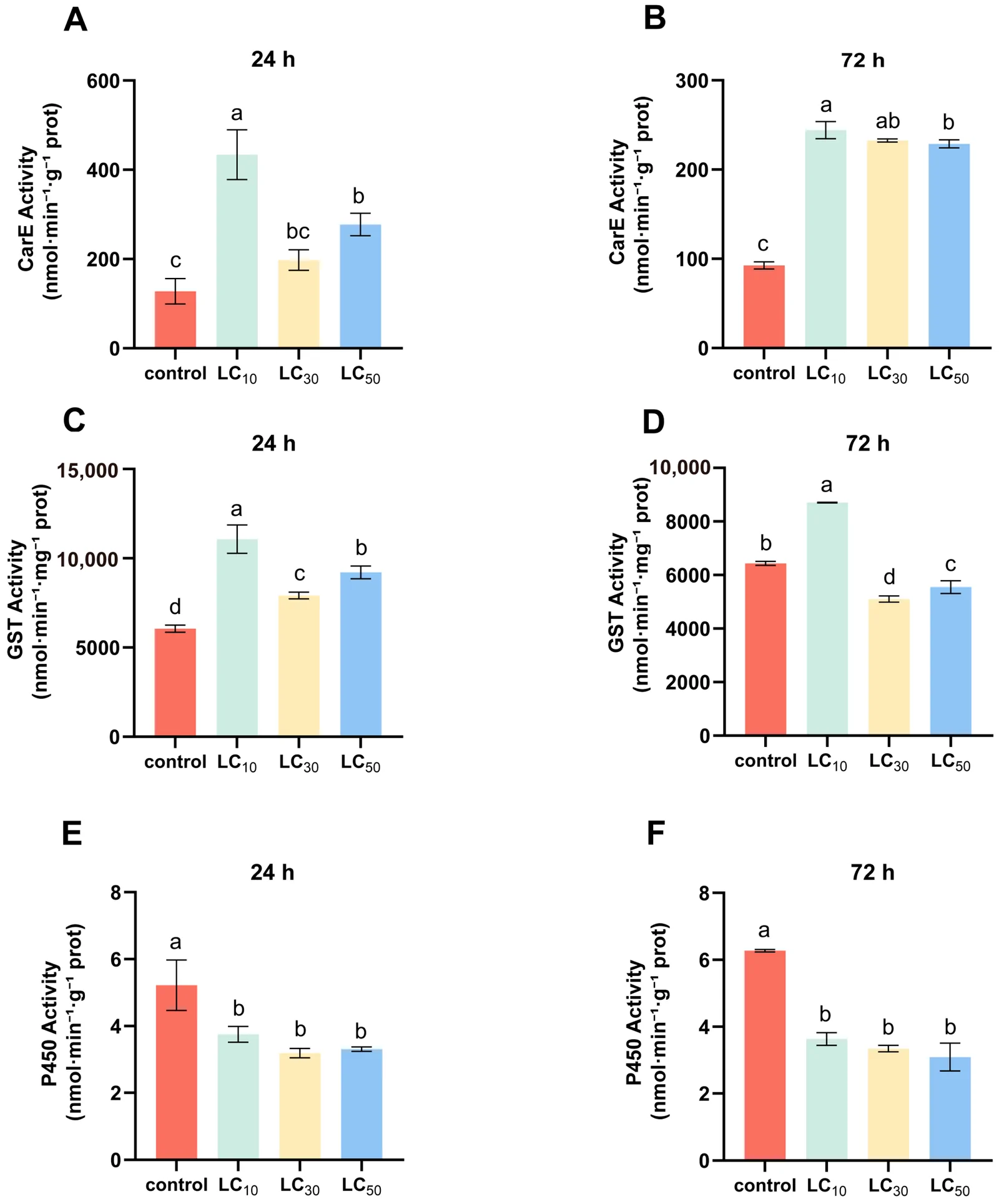
Effects of broflanilide exposure on detoxification enzyme activities in third-instar larvae of *Helicoverpa armigera*. (**A**,**B**) Carboxylesterase (CarE) activity at 24 h and 72 h after treatment, respectively. (**C**,**D**) Glutathione S-transferase (GST) activity at 24 h and 72 h after treatment, respectively. (**E**,**F**) P450 activity at 24 h and 72 h after treatment, respectively. Data are presented as mean ± SE of three biological replicates. Different lowercase letters indicate significant differences among treatments at the same sampling time according to Tukey’s honestly significant difference (HSD) test (*p* < 0.05).

The activities of CarE, GST and P450 in third-instar larvae under different broflanilide treatments are shown in Figure 2.

CarE activity increased after broflanilide exposure at both 24 and 72 h (Figure 2A,B). At 24 h, the highest CarE activity was observed in the LC_10_ group, reaching approximately 3.40-fold that of the control (*p* < 0.05). CarE activities in the LC_50_ groups were also significantly higher than that of the control, but no significant difference was observed between the LC_30_ treatment and the control. At 72 h, CarE activity decreased relative to the corresponding 24 h levels in LC_10_ and LC_50_ groups, but all treatments remained higher than the control level.

GST activity was significantly higher in all broflanilide-treated groups than in the control at 24 h, with the highest activity observed in the LC_10_ group (Figure 2C,D; *p* < 0.05). At 72 h, GST activity in the LC_10_ group remained higher than that of the control, whereas GST activity in the LC_30_ and LC_50_ groups decreased to 79.30% and 86.24% of the control level, respectively. GST activity differed among exposure concentrations and sampling times.

In contrast to CarE and GST, P450 activity was significantly lower in the broflanilide-treated groups than in the control (Figure 2E,F). At 24 h, P450 activity was significantly lower in all treatment groups than in the control (*p* < 0.05). At 72 h, P450 activity decreased further, reaching approximately 49.31%~57.92% of the control level, with no significant differences among the broflanilide concentrations.

Overall, CarE and GST activities generally increased after broflanilide exposure, whereas P450 activity decreased.

### 3.3. Gut Microbiota Alterations

#### 3.3.1. Alpha Diversity Analysis

**Figure 3:**
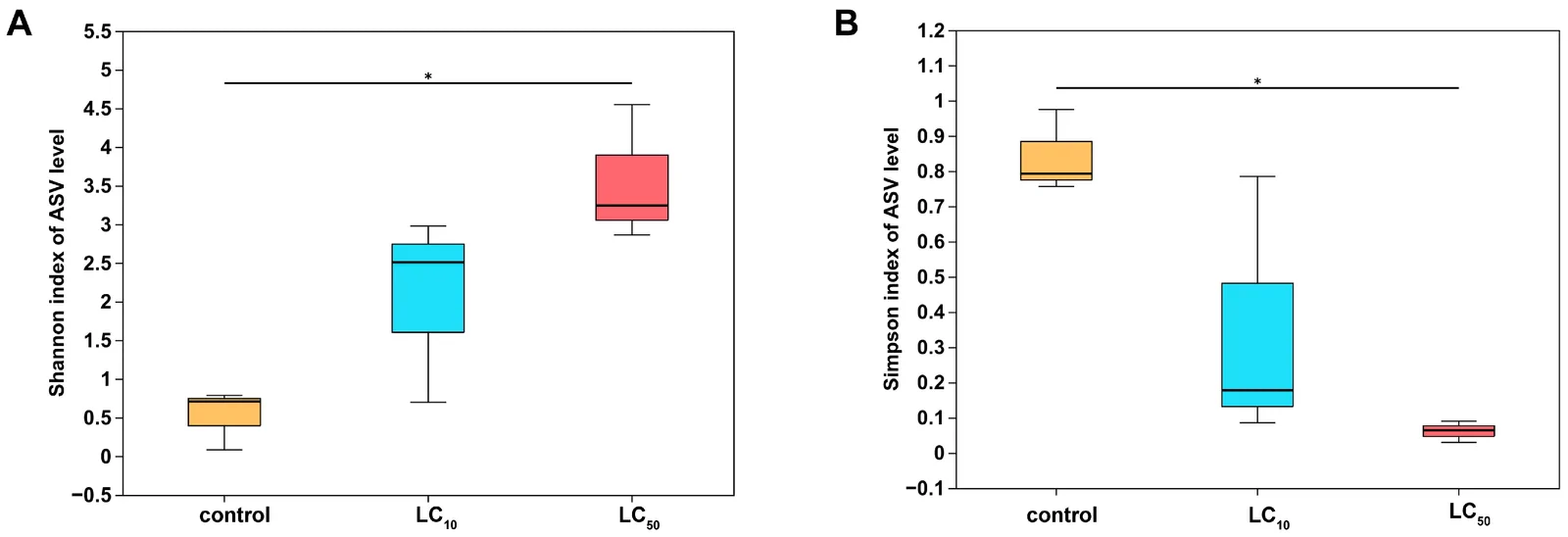
Effects of broflanilide exposure on gut bacterial alpha diversity in *Helicoverpa armigera*. Alpha diversity was assessed using the Shannon (**A**) and Simpson (**B**) indices. Data are shown as box plots, with the central line indicating the median and boxes representing the interquartile range. Significant pairwise differences are indicated by asterisks based on one-way ANOVA followed by Games–Howell post hoc tests with FDR correction (*p* < 0.05).

The Shannon and Simpson indices differed among treatments (Figure 3). Compared with the control, the LC_50_ group showed a significantly higher Shannon index and a significantly lower Simpson index (FDR-corrected *p* < 0.05). The LC_10_ group showed intermediate values for both indices, but no significant difference was indicated compared with the control.

#### 3.3.2. Beta Diversity Analysis

**Figure 4:**
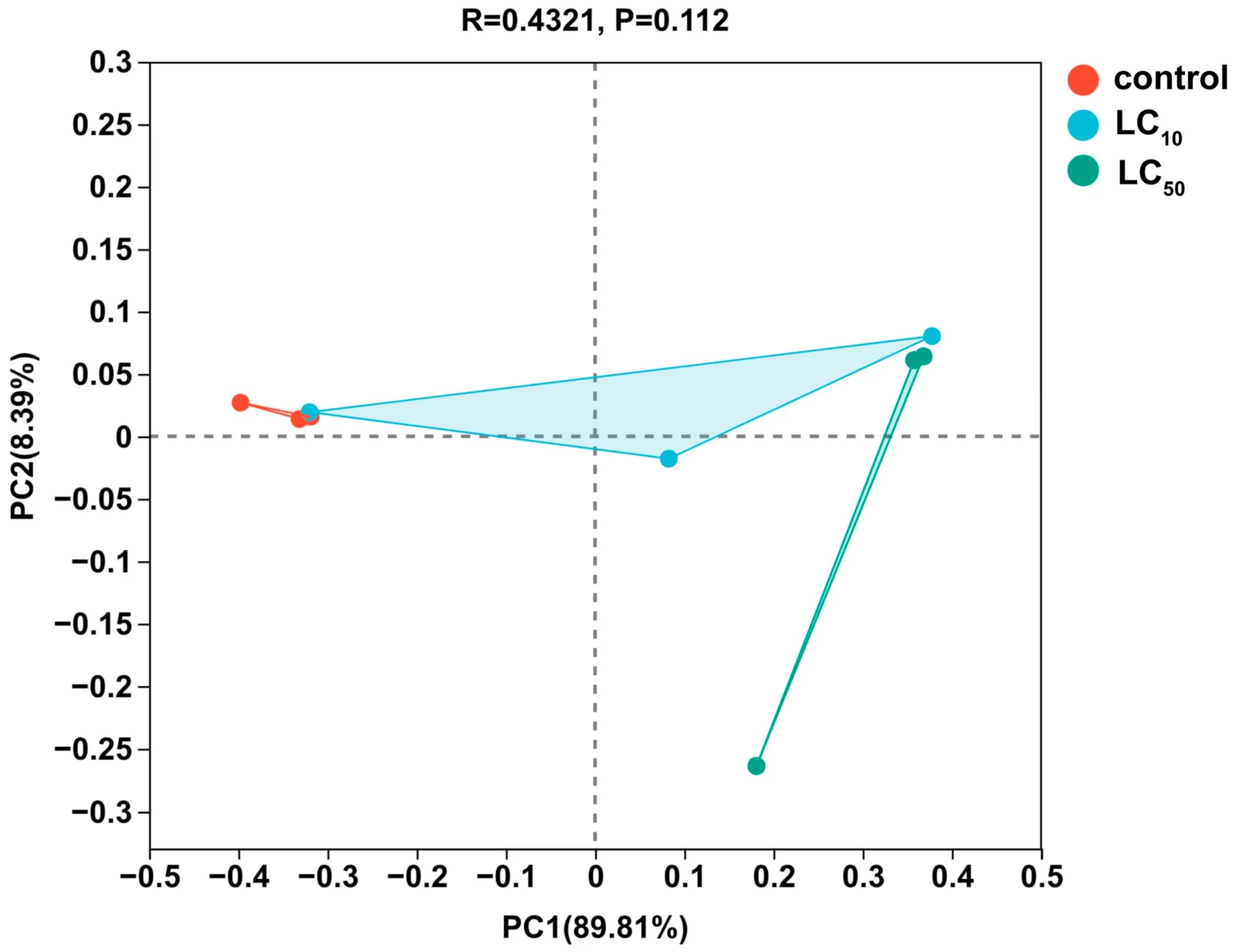
Principal coordinate analysis (PCoA) of gut bacterial communities in *Helicoverpa armigera* based on weighted UniFrac distances following broflanilide exposure. Each point represents one biological replicate. Different colors indicate different treatments (control, LC_10_, and LC_50_). Ellipses represent the 95% confidence intervals of each group.

Principal coordinate analysis (PCoA) based on weighted UniFrac distances showed that PC1 and PC2 explained 89.81% and 8.39% of the total community variation, respectively (Figure 4). ANOSIM detected no significant difference in bacterial community structure among treatments (*R* = 0.4321, *p* = 0.112).

#### 3.3.3. Community Composition Analysis

**Figure 5:**
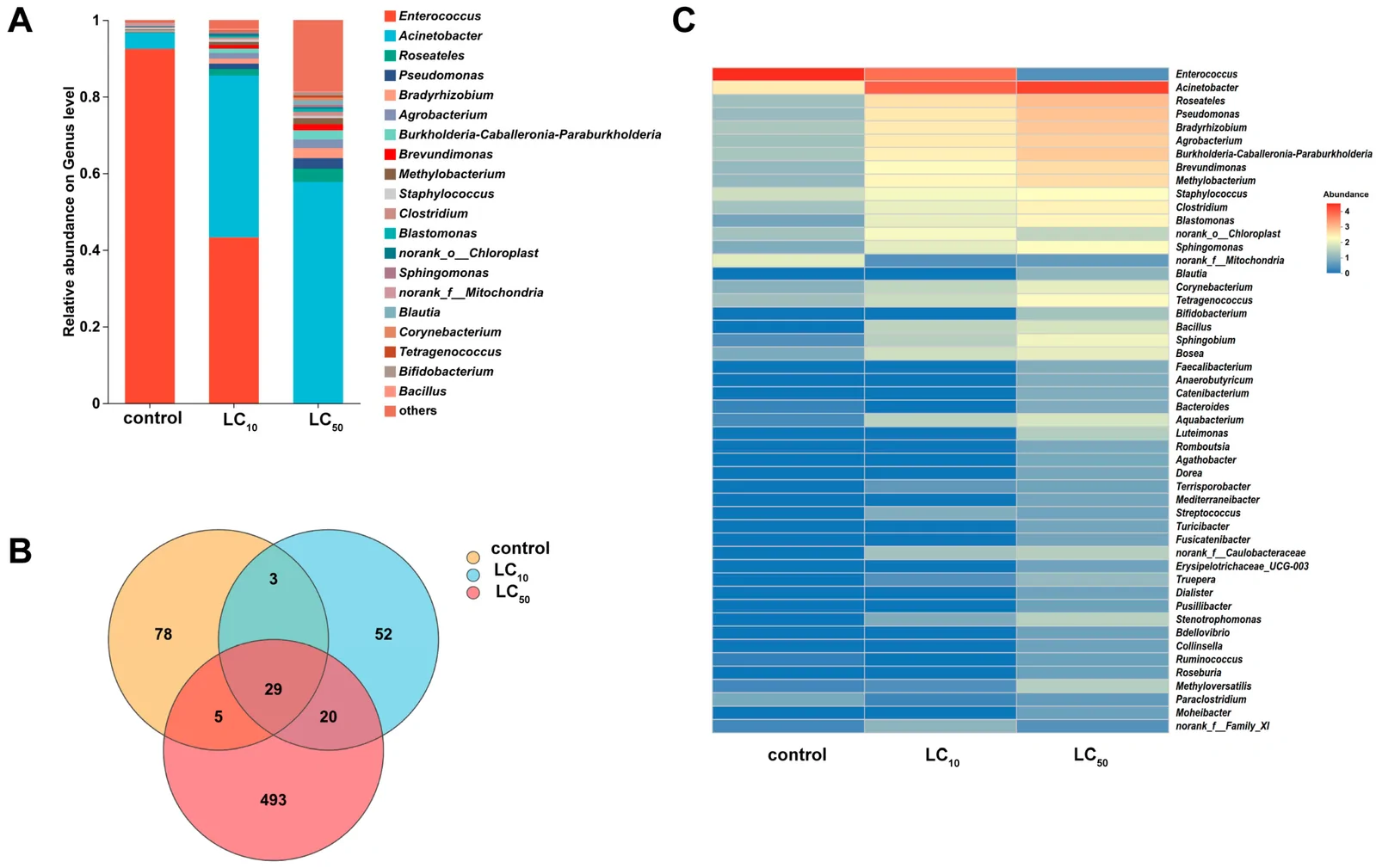
Gut bacterial community composition of *Helicoverpa armigera* after broflanilide exposure. (**A**) Relative abundance of dominant bacterial genera at the genus level in control, LC_10_, and LC_50_ treatment groups. Only the top 20 genera are shown, and the remaining taxa are grouped as “Others”. (**B**) Venn diagram showing shared and unique ASVs among control, LC_10_, and LC_50_ groups. (**C**) Heatmap of bacterial community composition at the genus level.

The gut bacterial community composition of *H. armigera* at the genus level is shown in Figure 5A. In the control group, *Enterococcus* was the dominant genus, with a relative abundance of 92.49%, followed by *Acinetobacter* (4.12%). The bacterial community in the LC_10_ group was generally similar to that of the control, with *Enterococcus* remaining the dominant genus. By contrast, the LC_50_ group showed differences in the relative abundance distribution of several bacterial genera. The relative abundance of *Acinetobacter* increased to 57.73%, making it the dominant genus, whereas the relative abundance of *Enterococcus* decreased. Several low-abundance genera also increased in relative abundance after LC_50_ exposure, including *Roseateles* (3.45%), *Pseudomonas* (2.72%), *Bradyrhizobium* (2.64%), *Burkholderia* (2.39%), *Agrobacterium* (2.30%), *Methylobacterium* (1.60%), *Brevundimonas* (1.49%) and *Clostridium* (1.11%).

Venn analysis at the ASV level showed that 29 ASVs were shared among the control, LC_10_ and LC_50_ groups (Figure 5B). The control, LC_10_ and LC_50_ groups contained 78, 52 and 493 unique ASVs, respectively. The LC_50_ group contained substantially more unique ASVs than the control. In contrast, the LC_10_ group showed relatively smaller changes.

The genus-level heatmap further illustrated differences in the relative abundance patterns of individual bacterial genera among treatments (Figure 5C). Both the control and LC_10_ groups were dominated by *Enterococcus*, whereas the LC_50_ group showed a higher abundance of *Acinetobacter* and changes in several low-abundance genera.

#### 3.3.4. Differential Taxa Analysis

**Figure 6:**
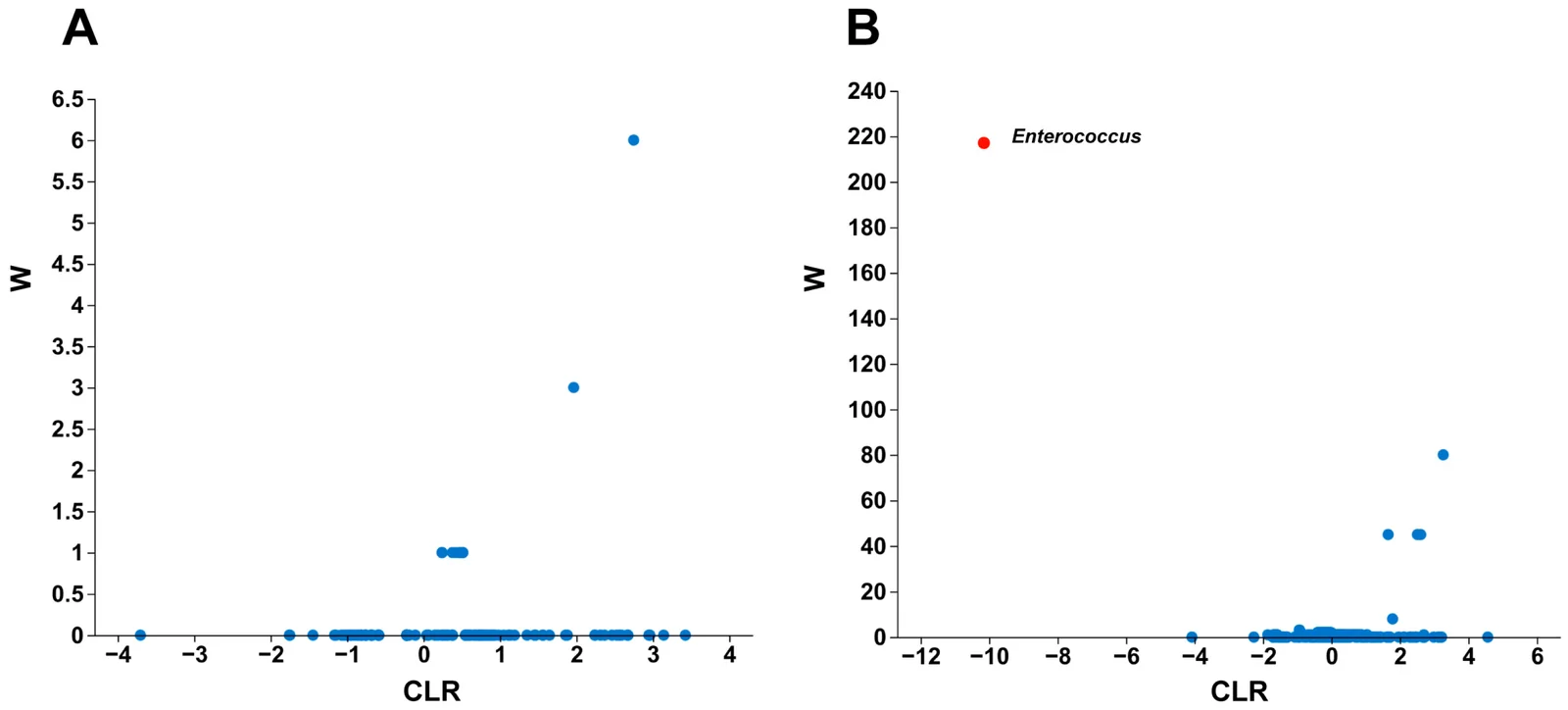
ANCOM analysis of differential gut bacterial genera in *Helicoverpa armigera* following broflanilide exposure. (**A**) Comparison between the LC_10_ treatment and control groups. (**B**) Comparison between the LC_50_ treatment and control groups. Each point represents a bacterial genus. Genera for which the ANCOM null hypothesis was rejected are highlighted in red and labeled; none were identified in (**A**).

Differential abundance of gut bacterial genera was further evaluated using ANCOM (Table S1; Figure 6). No bacterial genera were identified as significantly different between the LC_10_ and control groups according to the ANCOM criterion (Figure 6A). In contrast, *Enterococcus* was identified as the only significantly different genus between the LC_50_ and control groups, with a CLR value of −10.1406 and a W value of 217 (Figure 6B; Table S1). The negative CLR value reflected a lower abundance of *Enterococcus* in the LC_50_ group than in the control group.

#### 3.3.5. Functional Prediction of Gut Microbiota

**Figure 7:**
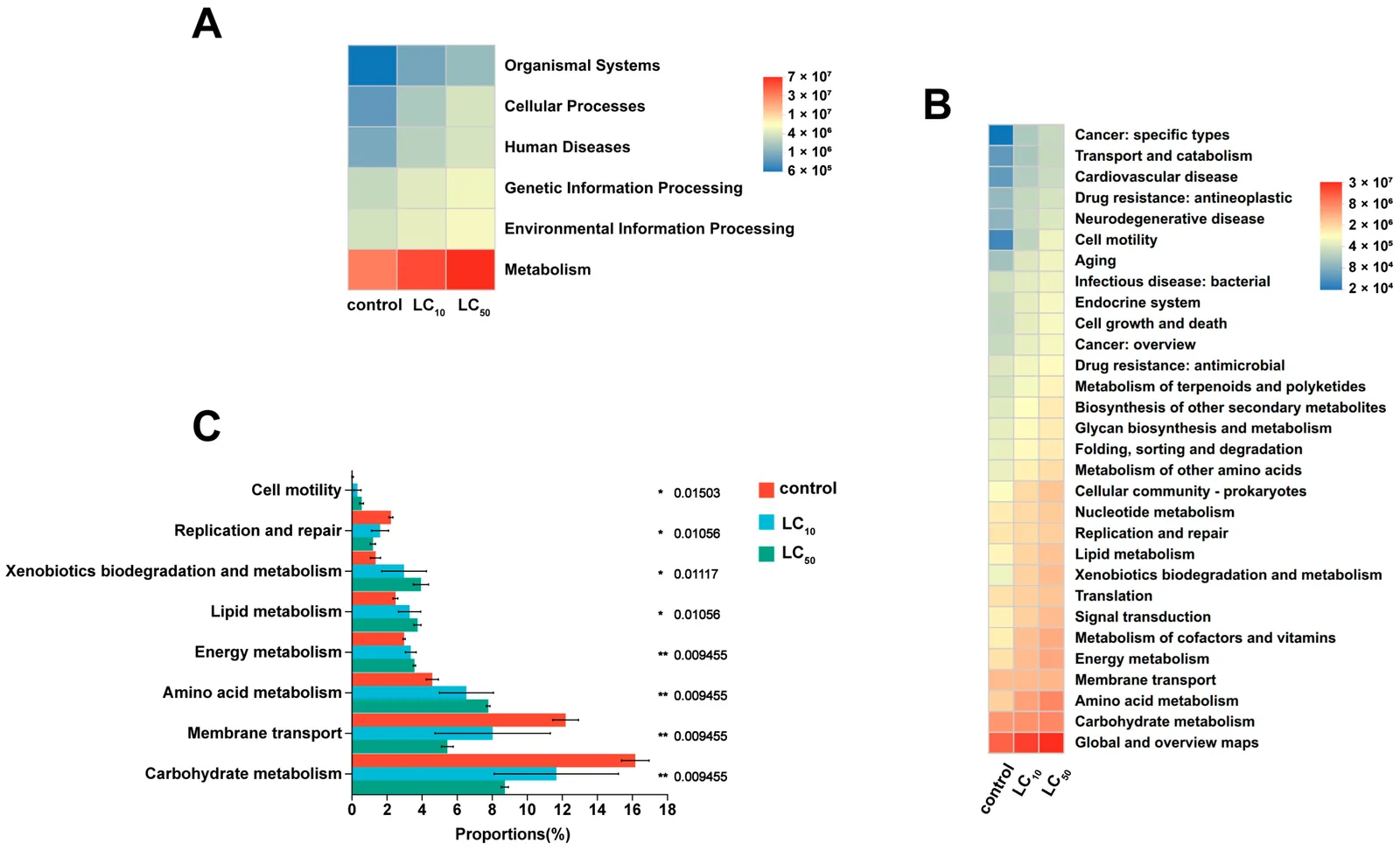
Functional prediction of gut microbiota in *Helicoverpa armigera* following broflanilide exposure based on PICRUSt2 analysis. (**A**) Relative abundance of KEGG Level 1 functional categories among different treatment groups. (**B**) Relative abundance of KEGG Level 2 functional categories among different treatment groups. (**C**) Differential KEGG Level 2 functional pathways of gut microbiota in *Helicoverpa armigera* following broflanilide exposure. Color intensity represents the relative abundance of predicted functional pathways. Significance levels are indicated by * (*p* < 0.05) and ** (*p* < 0.01).

Predicted functional profiles of the gut microbiota were inferred using PICRUSt2 based on KEGG pathways (Figure 7A,B). At KEGG Level 1, metabolism-related functions represented the largest proportion across all treatment groups. Compared with the control, the predicted relative abundances of Metabolism, Genetic Information Processing and Environmental Information Processing showed increasing trends in the LC_10_ and LC_50_ groups.

At KEGG Level 2, Carbohydrate Metabolism, Amino Acid Metabolism and Membrane Transport were among the most abundant predicted functional categories. Compared with the control, the predicted relative abundances of Xenobiotics Biodegradation and Metabolism and Drug Resistance: Antimicrobial increased in the LC_10_ and LC_50_ groups. Lipid Metabolism and Metabolism of Cofactors and Vitamins also showed increasing trends after broflanilide exposure (Figure 7B).

Differential analysis of predicted KEGG Level 2 pathways showed that broflanilide exposure was associated with changes in the predicted functional profiles (Figure 7C). Compared with the control, broflanilide-treated groups showed changes in pathways related to carbohydrate metabolism, membrane transport, amino acid metabolism, energy metabolism, lipid metabolism, xenobiotics biodegradation and metabolism, replication and repair, and cell motility. Among these pathways, xenobiotics biodegradation and metabolism, lipid metabolism, amino acid metabolism and energy metabolism tended to increase after broflanilide exposure, whereas carbohydrate metabolism and membrane transport showed decreasing trends. These results indicate that broflanilide exposure may influence the functional potential of gut microbial communities; however, these predicted pathways do not directly demonstrate broflanilide degradation or specific metabolic functions, which require further experimental validation.

## 4. Discussion

This study evaluated the lethal and sublethal effects of broflanilide on *H. armigera* and examined the associated physiological and gut microbial responses. Our previous research demonstrated that broflanilide exhibited high insecticidal activity against *H. armigera* under laboratory and field conditions [23]. The present study further revealed intergenerational effects on offspring fitness, together with changes in detoxification enzyme activities and specific gut bacterial taxa.

Parental exposure to broflanilide affected fitness-related traits in both the F_0_ and F_1_ generations, but the response patterns differed between exposure concentrations and generations. In the F_0_ generation, LC_30_ exposure primarily prolonged the duration of the fifth and sixth larval instars, whereas LC_50_ exposure reduced larval survival, adult emergence and reproductive output and increased adult malformation. In the F_1_ generation, parental LC_50_ exposure prolonged early larval development and markedly reduced larval survival, whereas pupal development was unaffected. Notably, adult emergence and fecundity were significantly reduced after parental exposure to both LC_30_ and LC_50_, indicating that reproductive performance in the offspring generation was sensitive even to the lower parental exposure concentration. The reduced intrinsic rate of increase and net reproductive rate in the F_1_ generation were likely associated with decreased adult emergence and fecundity, while lower larval survival and prolonged development under LC_50_ exposure may have further constrained population growth. Similar population-level effects have been reported for *Spodoptera litura* exposed to broflanilide [11]. Together, these findings suggest that broflanilide can suppress *H. armigera* population growth through acute toxicity as well as fitness costs expressed in surviving parents and their offspring [29,30]. 

Notably, F_1_ individuals showed reduced development and reproductive performance even though they were not directly exposed to broflanilide. This indicates that parental exposure can affect offspring fitness in *H. armigera* [31,32]. Previous studies have suggested that pesticide exposure may influence offspring performance through physiological regulation, altered energy allocation or microbiome-associated changes [33]. Our results are consistent with these findings, but the underlying mechanisms remain unclear. Because only the F_1_ generation was examined, these effects should be interpreted as intergenerational rather than fully multigenerational or transgenerational.

The detoxification enzyme assays showed distinct response patterns among enzyme systems. CarE activity increased after broflanilide exposure [34], whereas GST activity showed an early induction followed by a decline at higher concentrations or later exposure time [35]. In contrast, P450 activity was generally inhibited. Cytochrome P450 monooxygenases are often involved in insect metabolic adaptation to xenobiotics [17,36]. However, P450 activity was not induced under the tested conditions, whereas CarE and GST activities showed distinct responses to broflanilide exposure. These contrasting activity patterns indicate differential short-term biochemical responses among the three enzyme systems. Nevertheless, enzyme activity alone cannot confirm a causal role in broflanilide detoxification. Further studies using enzyme inhibitors, gene expression analysis, transcriptomics or metabolomics are needed to clarify the specific roles of these detoxification systems [37,38].

Gut microbiota analysis suggested that broflanilide exposure, particularly at LC_50_, was associated with changes in the abundance of specific bacterial taxa, although the overall bacterial community structure was not significantly altered. Insects rely on gut microbiota for nutrient metabolism, immune regulation and xenobiotic tolerance [39,40], and some symbiotic bacteria can contribute directly or indirectly to pesticide detoxification [20,41,42]. The pronounced decrease in *Enterococcus* under LC_50_ exposure indicates that this genus may be particularly responsive to broflanilide-associated changes in the gut microbiota. PICRUSt2 analysis further suggested potential increases in predicted pathways involved in xenobiotic biodegradation and metabolism [43]. However, these functional profiles were inferred from 16S rRNA amplicon data and should be regarded as predicted functional shifts rather than direct evidence of pesticide degradation. Metagenomic sequencing, bacterial isolation and functional validation experiments are required to determine whether these bacterial taxa contribute to broflanilide metabolism or host tolerance [27].

Overall, this study provides evidence that broflanilide affects *H. armigera* at multiple biological levels, including population fitness, detoxification enzyme activity and gut microbial responses. These findings improve our understanding of the ecological and physiological effects of meta-diamide insecticides. Future work should combine molecular and microbial functional analyses to clarify the roles of detoxification enzymes and gut bacteria in the physiological responses of *H. armigera* to broflanilide exposure [44].

## 5. Conclusions

This study assessed the lethal and sublethal effects of broflanilide on *H. armigera*. Parental exposure to LC_30_ and LC_50_ broflanilide prolonged larval development, reduced survival, adult emergence and fecundity, and decreased the intrinsic rate of increase (*r*) and net reproductive rate (*R*_0_) in the F_1_ generation. These results indicate that broflanilide can suppress population growth through both direct toxicity and offspring fitness costs.

Broflanilide exposure also altered detoxification enzyme activities, with increased CarE activity, time-dependent GST responses and inhibited P450 activity. In addition, although LC_50_ exposure did not significantly alter the overall gut bacterial community structure, it significantly reduced the relative abundance of *Enterococcus* and was associated with enrichment trends in predicted pathways related to xenobiotic metabolism. These findings suggest that detoxification metabolism and changes in specific gut bacterial taxa may be associated with the physiological response of *H. armigera* to broflanilide, although further functional validation is required.

Taken together, these results provide a scientific basis for the rational use of broflanilide in *H. armigera* management.

## Figures and Tables

**Table 1:** Effects of LC_30_ and LC_50_ broflanilide exposure on biological parameters of the F_0_ generation of *Helicoverpa armigera*.

Biological Parameters	Control	LC_30_	LC_50_
Duration of fifth and sixth larval instars (d)	5.92 ± 0.18 b	7.04 ± 0.14 a	7.39 ± 0.09 a
Larval survival (%)	90.80 ± 2.06 a	68.74 ± 8.74 a	39.00 ± 6.97 b
Pupal duration (d)	13.63 ± 0.48 a	13.31 ± 0.16 a	13.67 ± 0.60 a
Pupal weight (mg)	405.70 ± 6.52 ab	400.66 ± 5.30 b	426.45 ± 5.73 a
Pupation rate (%)	97.62 ± 2.38 a	87.20 ± 7.63 a	93.64 ± 3.19 a
Adult emergence rate (%)	100.00 ± 0.00 a	87.95 ± 4.79 ab	78.52 ± 6.46 b
Adult malformation rate (%)	5.13 ± 2.56 b	4.64 ± 1.41 b	15.88 ± 0.79 a
Fecundity (eggs/female)	2299.38 ± 271.52 a	2024.00 ± 129.03 ab	1557.50 ± 190.14 b
Oviposition period (d)	12.22 ± 0.11 a	11.93 ± 1.09 a	8.06 ± 0.34 b

Values are presented as mean ± SE. Different lowercase letters within the same row indicate significant differences among treatments according to Tukey’s honestly significant difference (HSD) test (*p* < 0.05). Footnotes also apply to Table 2.

**Table 2:** Effects of LC_30_ and LC_50_ broflanilide exposure on biological parameters of the F_1_ generation of *Helicoverpa armigera*.

Biological Parameters	Control	LC_30_	LC_50_
Duration of first instar (d)	2.04 ± 0.02 b	2.53 ± 0.29 ab	3.68 ± 0.43 a
Duration of second instar (d)	2.16 ± 0.14 b	2.22 ± 0.16 b	2.94 ± 0.43 a
Duration of third instar (d)	2.07 ± 0.02 b	2.27 ± 0.07 b	3.49 ± 0.09 a
Duration of fourth instar (d)	2.24 ± 0.01 b	2.30 ± 0.05 b	3.62 ± 0.24 a
Duration of fifth and sixth instars (d)	5.69 ± 0.07 a	5.76 ± 0.21 a	5.22 ± 0.27 a
Total larval duration (d)	14.21 ± 0.09 b	15.08 ± 0.62 b	18.95 ± 1.25 a
Larval survival (%)	84.00 ± 4.00 a	75.57 ± 11.87 a	31.19 ± 2.56 b
Pupal duration (d)	12.37 ± 0.25 a	13.15 ± 0.45 a	13.03 ± 0.22 a
Pupal weight (mg)	405.36 ± 3.44 a	414.77 ± 8.94 a	406.13 ± 9.18 a
Pupation rate (%)	100.00 ± 0.00 a	98.04 ± 1.96 a	93.27 ± 3.42 a
Adult emergence rate (%)	97.22 ± 2.78 a	65.28 ± 1.39 b	59.32 ± 1.63 b
Adult malformation rate (%)	2.38 ± 2.38 b	8.75 ± 2.29 b	19.77 ± 3.10 a
Fecundity (eggs/female)	2117.67 ± 162.99 a	1546.00 ± 139.65 b	1243.63 ± 285.90 c
Oviposition period (d)	11.55 ± 0.40 a	10.11 ± 0.29 ab	8.67 ± 0.84 b

**Table 3:** Life-table parameters of the F_1_ generation of *Helicoverpa armigera* after parental exposure to broflanilide.

Treatments	*r* (Day^−1^)	*λ* (Day^−1^)	*R*_0_ (Offspring/Individual)	*T* (Days)	*DT* (Days)
Control	0.1862 ± 0.0047 a	1.2046 ± 0.0057 a	758.91 ± 117.80 a	35.63 ± 0.24 b	3.72
LC_30_	0.1463 ± 0.0123 b	1.1576 ± 0.0142 b	215.72 ± 81.59 b	36.72 ± 0.71 b	4.74
LC_50_	0.1261 ± 0.0088 b	1.1344 ± 0.0100 b	188.63 ± 59.35 b	41.56 ± 0.94 a	5.50

Values of *r*, *λ*, *R*_0_, and *T* are presented as mean ± SE. *DT* is presented as a point estimate and was calculated as *DT* = ln(2)/*r*. *r*, intrinsic rate of increase; λ, finite rate of increase; *R*_0_, net reproductive rate; *T*, mean generation time; and *DT*, doubling time. Different lowercase letters within the same column indicate significant differences among treatments according to paired bootstrap tests (*p* < 0.05).

## Data Availability

The data presented in this study are available from the corresponding author upon reasonable request.

## Supplementary Materials

The following supporting information can be downloaded at: https://www.mdpi.com/article/10.3390/insects17080878/s1, Table S1. Representative bacterial genera identified by ANCOM analysis in the gut microbiota of *Helicoverpa armigera* following broflanilide exposure.
