# Supplementary material for: Parental Broflanilide Exposure Impairs Offspring Fitness and Alters Detoxification Enzymes and Gut Microbiota in Helicoverpa armigera
Source: Insects. 2026 Aug 21;17(8):878. doi: 10.3390/insects17080878 (PMC13512918; doi:10.3390/insects17080878)
Supplement: Supplementary file 1 [file insects-17-00878-s001.zip › insects-4475461-supplementary.pdf]

Table S1. Representative bacterial genera identified by ANCOM analysis in the gut microbiota of *Helicoverpa armigera* following broflanilide exposure.

Treatment	Feature	CLR	W	Reject null hypothesis
LC_{10}	norank_f__*Caulobacteraceae*	2.7489	6	FALSE
	Stenotrophomonas	1.9642	3	FALSE
	Parachlamydia	0.5129	1	FALSE
	Symbiobacterium	0.4292	1	FALSE
LC_{50}	*Enterococcus*	-10.1406	217	TRUE
	Bacillus	3.2648	80	FALSE
	norank_f__*Caulobacteraceae*	2.5129	45	FALSE
	Novosphingobium	1.6565	45	FALSE
	Stenotrophomonas	2.6066	45	FALSE

Representative bacterial genera with relatively high ANCOM W values are shown. CLR represents the difference in the mean centered log-ratio-transformed abundance between the broflanilide-treated and control groups. Positive CLR values indicate a higher abundance in the treatment group, whereas negative CLR values indicate a lower abundance. The W value represents the number of pairwise log-ratio tests for which the null hypothesis was rejected for a given genus. "TRUE" indicates that the genus met the ANCOM significance criterion, whereas "FALSE" indicates that it did not.
